# Supplementary material for: The effects of age and central field loss on maintaining balance control when stepping up to a new level under time-pressure
Source: PeerJ. 2023 Feb 20;11:e14743. doi: 10.7717/peerj.14743 (PMC9948744; doi:10.7717/peerj.14743)
Supplement: Supplemental Information 2 — Kinematic analysis of obstacle negotiation in the no-pressure and time-pressure conditions. This supplementary analysis demonstrates the effects of age and vision loss on obstacle negotiation under time-pressure. [file peerj-11-14743-s002.docx]

**KINEMATIC ANALYSIS OF OBSTACLE NEGOTIATION**

**Movement kinematics**

The included movement kinematics for obstacle negotiation are similar to a previous article that investigated the effects of time-pressure in participants with (simulated) vision loss (Zult et al., 2019). This movement kinematics have previously been identified as important for the assessment of obstacle negotiation (Timmis & Pardhan, 2012; Timmis & Buckley, 2012; Rhea & Rietdyk, 2011). The following kinematic variables were examined:

1. Vertical clearance height of the toe at the point of crossing the obstacle. The vertical toe clearance height was calculated as the vertical distance between the toe maker and the obstacle at the instant of obstacle crossing.
2. Horizontal crossing velocity of the toe at the point of obstacle crossing. The horizontal crossing velocity was calculated from the toe marker at the instant of obstacle crossing.
3. Penultimate and final foot placement before crossing the obstacle – horizontal distance between the toe and the obstacle. Foot placement was calculated as the horizontal distance between the toe marker and the obstacle at 0.01 s (1 frame) before toe off (i.e., the instant when the resultant velocity of the foot’s toe marker first increased more than 0.9 m/s for ten consecutive frames).
4. Lead limb single support time – swing time of the lead limb during obstacle crossing whereby only the trail limb is in contact with the ground (the trail limb has not crossed the obstacle yet).
5. Trail limb single support time – swing time of the trail limb during obstacle crossing whereby only the lead limb is in contact with the ground (the lead limb has already crossed the obstacle).

**Statistical analysis**

Data in text and tables are expressed as mean ± SD. The statistical analysis was performed using SPSS version 24. The Kolmogorov-Smirnov test was used to check normality and each kinematic variable was normally distributed. A group (AMD, older normals, young normals) by condition (no-pressure, time-pressure) mixed ANOVA was performed to test for differences in movement kinematics. Significant *F* values from the ANOVAs were subjected to a LSD post hoc pairwise comparison to determine the means that were different. The level of significance (α) was set at *p* < 0.05. Effect sizes were calculated using Cohen’s *d*.

**Results**

*Vertical toe clearance height.* Table 1 shows the vertical clearance heights for the lead and trail toe. The vertical clearance height of the lead toe did not show a significant group effect (F_2, 21_ = 0.2, p = 0.836), condition effect (F_1, 21_ = 3.2, p = 0.088), or group by condition interaction (F_2, 21_ = 0.7, p = 0.499). The clearance height of the trail toe also did not show a significant group (F_2, 21_ = 0.380, p = 0.688), condition (F_1, 21_ = 2.0, p = 0.170), or interaction effect (F_2, 21_ = 2.6, p = 0.100).

*Horizontal crossing velocity.* Table 1 shows the horizontal crossing velocity of the lead and trail toe. The horizontal crossing velocity of the lead toe showed a significant main effect of group (F_2, 21_ = 10.3, p = 0.001) and condition (F_1, 21_ = 65.2, p < 0.001) (Table 1). Post hoc testing for the group effect showed that young visual normals had a 19-42% faster horizontal crossing velocity than the older visual normals and AMD participants (both p ≤ 0.023, d ≥ 0.73). The horizontal crossing velocity of the lead toe was not significantly different between older visual normals and AMD participants (p = 0.051). The condition effect revealed that the crossing velocity of the lead toe was 33% faster in the time-pressure than no-pressure condition (p < 0.001, d = 1.21). No significant group by condition interaction was observed (F_2, 21_ = 3.4, p = 0.052).

The horizontal crossing velocity of the trail toe showed a group (F_2, 21_ = 7.4, p = 0.004), condition (F_1, 21_ = 58.4, p < 0.001), and interaction effect (F_2, 21_ = 4.7, p = 0.021) (Table 1). Post hoc testing for the interaction effect showed that in the no-pressure condition, young visual normals had a 22% faster horizontal crossing velocity than AMD participants (p = 0.034, d = 1.03). In the time-pressure condition, young visual normals demonstrated a 28-52% faster horizontal crossing velocity compared to older visual normals and AMD participants (both p ≤ 0.019, d ≥ 1.24). No other significant differences were observed between groups (p ≥ 0.157). On comparing between conditions for each group, all three groups showed a 25-56% higher horizontal crossing velocity in the time-pressure vs. no-pressure condition (all p ≤ 0.022, d ≥ 0.88).

*Foot placement.* Table 1 shows the results for penultimate and final foot placement. Penultimate foot placement showed a significant main effect of group (F_2, 21_ = 4.7, p = 0.020) and condition (F_1, 21_ = 8.2, p = 0.009) (Table 1). Post hoc testing for the group effect showed that young visual normals placed their foot 31% further away from the obstacle than AMD participants (p = 0.006, d = 0.84). No other significant differences were observed between groups (all p ≥ 0.083). The condition effect revealed that the foot was placed 15% further away from the obstacle in the time-pressure than no-pressure condition (p = 0.009, d = 0.57). No significant group by condition interaction was observed (F_2, 21_ = 1.2, p = 0.309).

Final foot placement did not show a significant group effect (F_2, 21_ = 0.4, p = 0.705), condition effect (F_1, 21_ = 4.0, p = 0.059), or group by condition interaction (F_2, 21_ = 0.1, p = 0.878).

*Single support time.* Table 1 demonstrates the single support times of the lead and trail limb during crossing. A main effect of condition was observed for the single support time of the lead limb (F_1, 21_ = 21.8, p < 0.001). The condition effect revealed that the single support time of the lead limb was 13% shorter in the time-pressure than no-pressure condition (p < 0.001, d = 1.07). No significant group effect (F_2, 21_ = 0.8, p = 0.452) and group by condition interaction was observed (F_2, 21_ = 0.9, p = 0.410).

A main effect of condition was also observed for the single support time of the trail limb (F_1, 21_ = 19.4, p < 0.001). The condition effect revealed that the single support time of the trail limb was 13% shorter in the time-pressure than no-pressure condition (p < 0.001, d = 0.95). No significant group effect (F_2, 21_ = 1.2, p = 0.313) and group by condition interaction was observed (F_2, 21_ = 2.1, p = 0.151).

Table 1. Obstacle negotiation kinematics of AMD participants, older visual normals and young visual normals in the two different conditions (mean ±SD).

| Variables | AMD participants (n=8) | |  | Older visual normals (n=8) | |  | Young visual normals (n=8) | | ANOVA |
| --- | --- | --- | --- | --- | --- | --- | --- | --- | --- |
|  | No-pressure | Time-pressure |  | No-pressure | Time-pressure |  | No-pressure | Time-pressure |  |
| Lead vertical toe clearance (m) | 0.18 (0.05) | 0.18 (0.04) |  | 0.17 (0.05) | 0.15 (0.04) |  | 0.18 (0.04) | 0.17 (0.05) | ns |
| Trail vertical toe clearance (m) | 0.16 (0.05) | 0.13 (0.04) |  | 0.12 (0.05) | 0.14 (0.04) |  | 0.15 (0.09) | 0.12 (0.03) | ns |
| Lead horizontal toe velocity (m/s) | 2.7 (0.6) | 3.4 (0.7) |  | 3.2 (0.5) | 4.1 (0.8) |  | 3.6 (0.3) | 5.1 (0.9) | G, C |
| Trail horizontal toe velocity (m/s) | 2.3 (0.3) | 2.9 (0.8) |  | 2.5 (0.4) | 3.5 (0.6) |  | 2.9 (0.6) | 4.4 (0.9) | G, C, GxC |
| Penultimate foot placement (m) | 0.72 (0.17) | 0.84 (0.24) |  | 0.86 (0.23) | 0.90 (0.12) |  | 0.92 (0.20) | 1.13 (0.15) | G, C |
| Final foot placement (m) | 0.22 (0.03) | 0.25 (0.08) |  | 0.23 (0.08) | 0.25 (0.07) |  | 0.23 (0.09) | 0.28 (0.06) | ns |
| Single support time lead foot (s) | 0.55 (0.09) | 0.48 (0.06) |  | 0.51 (0.05) | 0.47 (0.06) |  | 0.53 (0.06) | 0.44 (0.05) | C |
| Single support time trail foot (s) | 0.53 (0.05) | 0.49 (0.06) |  | 0.57 (0.12) | 0.46 (0.03) |  | 0.50 (0.05) | 0.45 (0.08) | C |

G, significant group effect (*p* < 0.05); C, significant condition effect (*p* < 0.05); GxC, significant group by condition interaction (*p* < 0.05); ns, no significant effect.

**Conclusions**

This supplementary analysis was performed to investigate the effects of age and vision loss on obstacle negotiation under time-pressure. The participants had to walk along a travel path that consisted of a 10 cm high obstacle followed by a floor-mounted force plate, reflecting a low curb to step-up onto. Differences in obstacle negotiation were observed for young visual normals compared to older visual normals and AMD participants. Young visual normal crossed the obstacle 19-52% faster than older visual normals and AMD participants, while no significant differences were observed between older visual normals and AMD participants. The slower crossing velocity for older visual normals and AMD participants is likely caused by the slower performance of the task. Performing the task under time-pressure resulted in changes in obstacle negotiation that were associated with walking faster (i.e., faster crossing velocity of the lead and trail foot, penultimate foot placement further away from the obstacle, and shorter single support times). The vision loss of AMD participants did not affect obstacle negotiation, which is in contrast to previous research in which participants with simulated blur (i.e., acute visual impairment) negotiated the obstacle more cautiously under time-pressure compared to those with normal vision (Zult et al., 2019). It might be that the AMD participants in the current study (i.e., chronic visual impairment) have adopted a visual search strategy that allow them to negotiate the obstacle like young and older visual normals. Future research should examine the visual search behaviour between visual normals and AMD participants when performing an obstacle course under no-pressure and time-pressure conditions.

**References**

Rhea CK, Rietdyk S. 2011. Influence of an unexpected perturbation on adaptive gait behavior*. Gait & posture*, 34:439-441.

Timmis MA, Buckley JG. 2012. Obstacle crossing during locomotion: visual exproprioceptive information is used in an online mode to update foot placement before the obstacle but not swing trajectory over it*. Gait & posture*, 36:160-162.

Timmis MA, Pardhan S. 2012. Patients with central visual field loss adopt a cautious gait strategy during tasks that present a high risk of falling*. Investigative ophthalmology & visual science*, 53:4120-4129.

Zult T, Allsop J, Timmis MA, Pardhan S. 2019. The effects of temporal pressure on obstacle negotiation and gaze behaviour in young adults with simulated vision loss*. Scientific reports*, 9:1-13.
